# Potential drug interactions in patients given antiretroviral therapy

**DOI:** 10.1590/1518-8345.1193.2832

**Published:** 2016-11-21

**Authors:** Wendel Mombaque dos Santos, Silvia Regina Secoli, Stela Maris de Mello Padoin

**Affiliations:** 1 MSc, RN, Empresa Brasileira de Serviços Hospitalares, Santa Maria, RS, Brazil; 2 PhD, Associate Professor, Escola de Enfermagem, Universidade de São Paulo, São Paulo, SP, Brazil; 3 PhD, Adjunct Professor, Departamento de Enfermagem, Universidade Federal de Santa Maria, Santa Maria, RS, Brazil

**Keywords:** Drug Interactions, Anti-HIV Agents, Drug Incompatibility

## Abstract

**Objective:**

to investigate potential drug-drug interactions (PDDI) in patients with HIV infection on antiretroviral therapy.

**Methods:**

a cross-sectional study was conducted on 161 adults with HIV infection. Clinical, socio demographic, and antiretroviral treatment data were collected. To analyze the potential drug interactions, we used the software Micromedex(r). Statistical analysis was performed by binary logistic regression, with a p-value of ≤0.05 considered statistically significant.

**Results:**

of the participants, 52.2% were exposed to potential drug-drug interactions. In total, there were 218 potential drug-drug interactions, of which 79.8% occurred between drugs used for antiretroviral therapy. There was an association between the use of five or more medications and potential drug-drug interactions (p = 0.000) and between the time period of antiretroviral therapy being over six years and potential drug-drug interactions (p < 0.00). The clinical impact was prevalent sedation and cardiotoxicity.

**Conclusions:**

the PDDI identified in this study of moderate and higher severity are events that not only affect the therapeutic response leading to toxicity in the central nervous and cardiovascular systems, but also can interfere in tests used for detection of HIV resistance to antiretroviral drugs.

## Introduction

HIV infection affects 36.9 million people worldwide, representing about 0.6% of the world's population. There are an estimated 1.6 million deaths yearly due to acquired immunodeficiency syndrome (AIDS)^1^. This disease causes a negative impact of multidimensional order into the lives of people. However, a great transformation occurred in the epidemiological profile with the emergence of highly active antiretroviral therapy (HARRT) ^(^ ^2^.

The use of HARRT, which can reduce the viral load to undetectable levels and raise the count of CD4^+^ T lymphocytes, resulted in the mortality reduction and increased survival rate of the infected individuals^2^. However, the success of HARRT is associated with the maintenance of a high rate of patient compliance and the prevention and management of drug-drug interactions (DDI)^3^.

DDI is defined as a clinical or pharmacological effect that results from the co-administration of medications, which alter a patient's response to treatment. DDI occurs when the action of one drug (object, substrate) is altered by the presence of another drug (precipitant, interacting drug)^4^. DDI represents one of the most frequent adverse drug events that result in hospitalization, increase of cardiovascular risk, and abandonment of treatment. These induce adverse events or reduce the therapeutic efficacy, particularly in individuals subjected to polypharmacy^5^.

Polypharmacy, combined with factors such as age, alcohol consumption, illicit drug use, and potentially interactive features of some antiretroviral drugs such as protease inhibitors and reverse transcriptase inhibitors but not nucleoside analogues increase the complexity of therapeutic management and the risks pertaining to DDI^4^ ^-^ ^7^.

Antiretroviral therapy (ART) agents represent one of the main therapeutic groups with the greatest potential for DDI. Both protease inhibitors and nucleoside analogues are substrates and modulators of the cytochrome P450 enzyme system^6^ ^-^ ^9^. International consensus and national guidelines for the management of patients undergoing HARRT, must be put in place to avoid hardships arising from DDI. Despite this, studies conducted in different countries indicate that the prevalence of DDI in the users of ART in outpatient context varies from 21.5% to 67.1%, depending on the age of individuals, the therapeutic classes involved, and the database used to analyze DDI^8^ ^-^ ^9^. Participants exposed to DDI showed reduced treatment adherence^9^.

Post-marketing surveillance reported the use of HARRT focused on the identification of potential drug-drug interactions (PDDI), particularly in Brazil, where there are over 405,000 individuals involved in the treatment and this surveillance can contribute to a better understanding and management of clinically relevant PDDI^10^. The term PDDI refers to the possibility of a particular medication altering the intensity of the pharmacological effects of another medication therefore increasing or decreasing the therapeutic effect and/or adverse reactions or the responses other than those originally stemming from the medications ^(^ ^10^.

In this context, it is fundamental that health professionals have knowledge regarding PDDI in people subjected to antiretroviral treatment, as the prescription must consider the characteristics of the drugs and especially the possibilities of these interactions. The scientific literature shows that few studies in the area are carried out by nurses, even though the routine of medications should occupy a strategic position leading to interactions in order to enable nurses to examine their daily work and interfere with medication routine geared to to prevent the occurrence of adverse reactions due to drug interactions. The objective of this study was to determine the prevalence of potential drug-drug interactions in patients with HIV infection undergoing HARRT and to identify the major PDDI in this group and associated factors.

## Methods

This cross-sectional study was performed at the infectious disease clinic of the University Hospital of Santa Maria/RS/Brazil during the period from January to June 2012. This clinic in Southern Brazil treats approximately 432 patients yearly. The study was approved by the Research Ethics Committee at the Federal University of Santa Maria, Brazil, under number CAAE 0332.0.243.000-11. Upon a participant's arrival, the Informed Consent Form was provided, with enough time to study it. Afterwards, they were given a chance to ask questions, and questionnaires were applied in a restricted way in order to maintain the confidentiality diagnosis of HIV infection, and the data submitted. Informed, written consent was given by the participants prior to data collection.

Study participants volunteered to form a convenience sample, determined using a list of registered patients at the clinic. Consecutively, the study comprised participants adults, infected with HIV, registered within a drug distribution unit, and monitored in an ambulatory infectious disease setting. We excluded individuals whose HARRT regimen was not included in their chart and were under treatment for tuberculosis, because the use of more drug interactions and in a limited period of treatment. The following parameters were used to calculate the sample size: population size, 432 individuals; type 1 error (α), 0.05; test power (1-ß), 0.90. According to these criteria, the sample size resulted in 179 participants.

The necessary information was obtained by interviews of the participants and referring to the medical records of patients. The dependent variable was PDDI in HARRT. For obtaining information about the medicines we asked the question, "Which antiretroviral drug did the participants use?" and for the use of drugs, we asked the question, "What are the other medicines that you use?"

There were two sets of independent variables, socio-demographic/clinical (age, gender, education, marital status, family income, time of diagnosis, drug consumption, alcohol intake, CD4^+^ T-cell counts, and viral load) and HARRT (adherence to HARRT, treatment time, polypharmacy, and the reporting of adverse reactions as a result of HARRT).

To evaluate the adherence to HARRT, we used the "questionnaire for the evaluation of adherence to ART in people with HIV/AIDS (CEAT-VIH)" translated and validated in Portuguese(11). We used the CAGE questionnaire to evaluate alcohol consumption(12). Polypharmacy was considered by the use of five or more medicines by the patient(13).

The medicines were initially classified by the anatomical therapeutic chemical (ATC) system of the World Health Organization, which divides substances into different groups according to the organ or system on which they act and their chemical, pharmacological, and therapeutic properties(14). The level five of this system, which represents chemicals, was used for identification of DDI. Using the first level of this system the following groups were found: Group J (Anti-infection For Systemic Use); Group N (Nervous System); Group M (Muscle-Skeletal System).

All co-administered medications were included in the PDDI analysis using the electronic database, Micromedex? Healthcare Series. This database allows to sort the DDI following gravity and effect. In addition, it provides a description of the clinical impacts of DDI.

Descriptive statistics were used for the submission of PDDI and Pearson's Chi-square, and Fisher's exact tests were used for the bivariate analysis of the data. Binary logistic regression was used to obtain the estimates of the odd's ratio (OR) and confidence intervals. P-values less than 0.05 were considered statistically significant.

## Results

The subjects included 161 participants undergoing HARRT, of which 52.2% (n = 84) were exposed to PDDI. The average viral load was 5658.89 ± 30020.70 copies/ml, and the average CD4^+^ T-cell counts was 476.17 ± 269.69 cells/µl. About 44% of the population had some opportunistic disease in the last year, however there were not present at the time of data collection.

The groups of individuals exposed to potential PDDI and unexposed to PDDI, showed no differences pertaining to gender, age, alcohol consumption, drug use, adherence to therapy, or adverse reaction reporting. The polypharmacy (p = 0.000) and time of treatment (p = 0.00) showed a significant association with the presence of DDI.

The average age in the group of individuals exposed to potential PDDI and unexposed to PDDI was 44.1 ± 10.5 years and 41.0 ± 10.3 years, respectively (range: 22-67 years). Among individuals exposed to PDDI, 7.1% were elderly, whereas among individuals unexposed to PDDI, 5.2% were elderly. There was statistically significant difference (p = 0.001) among the mean of drugs used in the PDDI group: the mean was 5.08 ± 0.92 and in the non-PDDI group was 4.01 ± 0.14. The average of ART was statistically significant (p = 0.001), individuals have used 4.68 ± 0.66 and 0.33 ± 3.87 respectively comparing PDDI group and non-PDDI group. The average treatment time in the PDDI group was 7.17 ± 4.43 years and in the non-PDDI group was 5.26 ± 4.29 years with a significant difference between the two groups (p = 0.001).

We identified 29 different medications used by individuals in this study. Approximately half (51.8%) of them were systemic anti-infection agents (Group J), 44.8% were directed for the nervous system (Group N), and 3.4% had action in the musculoskeletal system (Group M).

The medicines of Group J included lamivudine (99.4%) and abacavir (77%) and zidovudine (77%), efavirenz (49.7%) and ritonavir (49.1%). Among the medicines used for ART, 40% and 33.4% were Reverse Transcriptase Inhibitors Nucleoside Analogues and protease inhibitors, respectively, and about a third of them were system substrates.

In Group N, the most-used medicines were diazepam (44.8%), amitriptyline (20.7%), clonazepam (17.2%), and fluoxetine (17.2%). Of these, 76.9% were inhibitors of the cytochrome P450 system.

There were 218 identified PDDI, of which 79.8% occurred among ART agents used in HARRT, 12.8% between HARRT and Group N, 4.2% between the drugs affecting the CNS (central nervous system), and 3.2% between alcohol and Group N. Among the participants who consumed alcohol, the most widely used medicines for ART were lamivudine (100%) and abacavir (79%), zidovudine (79%), efavirenz (53.5%), ritonavir (46.5%) and lopinavir (25.6%).

In the group with PDDI, the average of interactions between ART per patient was 2.07 ± 0.75 and among all therapeutic classes was 2.63 ± 1.42.

The PDDI could result in the changes in the plasma concentration of medicines (73.9%) and reduce the therapeutic efficacy of medicines (62.7%). Among the clinical manifestations cardiotoxicity appeared in 18%. Table 1 shows the risk factors to the potential drug interactions.

**Table 1:**
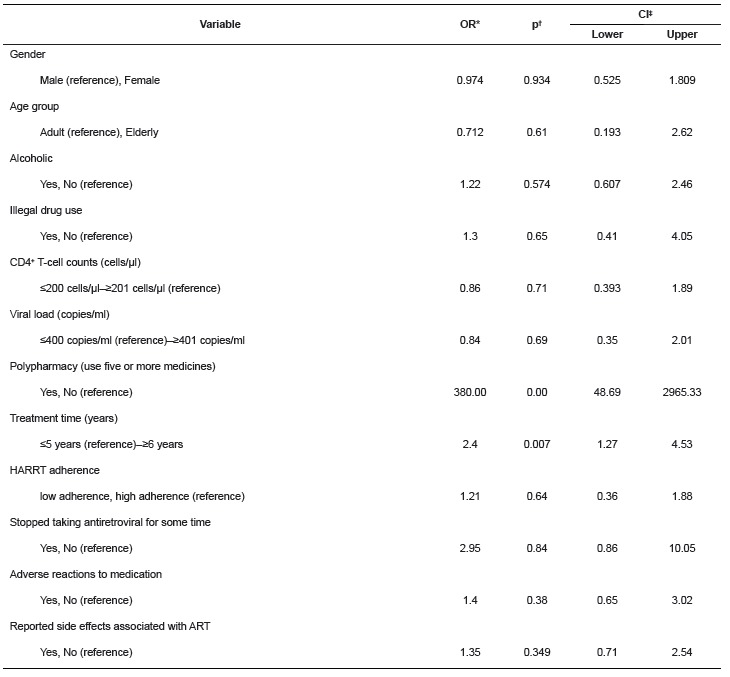
Logistical regression of the factors associated with the potential drug interactions. Santa Maria, RS, Brazil, 2012

*OR, *odds ratio*;

† Binary logistic regression

‡ CI, Confidence Interval

It was observed that there was predominance in the group with PDDI treatment time exceeding 6 years in all the levels of gravity. There was a statistically significant difference (p < 0.01) between the groups of patients with treatment of up to 5 years and with 6 years or more and the presence of PDDI.

The DDIs of HARRT are shown in Table 2. Ritonavir was the medicine used for ART with the most DDI (64.9%). Among the medicines affecting the CNS, diazepam (8.2%) had the most PDDI.

**Table 2:** The ten potential drug interactions between antiretroviral with its severity and documentation changes. Santa Maria, RS, Brazil, 2012

Drug 1	Drug 2	%	Gravity	Evidence	Effects*
Ritonavir Lopinavir	Zidovudine	39	Moderate	Good	reduced zidovudine bioavailability
Ritonavir Lopinavir	Tenofovir	17.4	Moderate	Good	increased tenofovir bioavailability
Atazanavir	Ritonavir	12.8	Moderate	Excellent	increased risk of PR interval prolongation
Bromazepam Diazepam Clonazepam	Ritonavir	6.4	Moderate	Good	increased risk of extreme sedation and confusion, increased clonazepam serum concentrations and potential toxicity, increased bromazepam plasma concentrations
Atazanavir	Tenofovir	4.6	Major	Excellent	decreased atazanavir concentrations/exposure and/or increased tenofovir concentrations
Darunavir	Ritonavir	2.3	Major	Good	decreased exposure to darunavir
Diazepam	Ethanol	2.3	Moderate	Fair	increased sedation
Darunavir	Tenofovir	1.8	Moderate	Excellent	increased tenofovir exposure
Diazepam	Fluoxetine	1.4	Minor	Good	higher serum concentrations of diazepam
Fluoxetine	Ritonavir	1.4	Major	Excellent	increased fluoxetine exposure; increased risk of QT-interval prolongation

*Electronic database of Micromedex^(r)^ Healthcare Series

Among patients with PDDI, 60.7% showed two PDDIs and 16.7% showed three PDDIs. About two out of ten individuals were exposed to four to nine PDDIs.

## Discussion

Despite the evidence of international and Brazilian guidelines for HARRT and the issue of interactions and potential adverse events associated with it^8^ ^-^ ^9^ ^,^ ^15^, the prevalence in this study (52.2%) was higher in comparison to other research conducted with adults for outpatient treatments in India (21.5%)^8^, the United Kingdom (27%)^9^, and England (35%)^9^. Possible explanation for this difference is the fact that this study looked at the PDDI between medicines of groups with high-potential interactions (antiretroviral, central nervous system, and ethanol). In addition, the overall average of drugs consumed and age of the participants in this investigation was higher compared to other studies.

The fact that the prescription of anti-retroviral drugs was held by Infectious disease specialists who manage specialty guidelines in daily life could have contributed to a reduced prevalence of PDDI. However, this is not what actually occurred, considering that the rate of drug interactions was high. Investigation found that 32.5% of physicians who prescribed ART lacked any kind of specialization, something that may influence the occurrence of DDI^16^.

The association between polypharmacy and PDDI was confirmed, a fact in line with other investigations that analyzed ART^9^. Polypharmacy is a risk factor in patients undergoing HARRT and relates particularly to those individuals who have a treatment regimen with at least a medication not belonging to the ART group, which can be aggravated by age^17^. Elderly patients have 51% probability of DDI and youngsters a 35% chance in the case of use of 6 and 7 medications, respectively^17^.

Each medicine added in the therapy increases the risk of adverse events by 10%, including DDI^17^. Despite the risk of DDI associated with polypharmacy, this strategy is critical in HIV-infected patients. The first line of initial treatment typically includes three ART, two Nucleoside analogues, and non-Nucleoside analogs. The first-line treatment consists of tenofovir, lamivudine, and efavirenz and the second-line treatment consists of protease inhibitor and nucleoside analogues^15^. In addition, in cases where there is a presence of opportunistic infections or co-morbidities, polypharmacy is mandatory^15^.

The HARRT exposure time equal to or above 6 years was associated to a 2.4 fold increase in PDDI. Throughout the treatment, there may be new ART and medicines of other classes (for example, drugs affecting the central nervous system) to handle psychiatric disorders such as anxiety and depression. These patients may also start recreational consumption of alcohol. There is evidence that depression affects about 21% of patients with HIV, and that individuals with this disorder are 19 times at greater risk of alcohol abuse^18^.

Accordingly, longer exposure to HARRT causes increased frequency of adverse reactions to medication. Evidence suggests that the incidence of adverse reactions to medication is about 50% in adults who receive HARRT in the outpatient setting^19^. The presence of adverse reactions to medication is 1.6 times higher in people with a CD4^+^ T-cell count below 200 cells/µl^20^. This may be more associated with the greatest number of drugs used by individuals than with a lower number of these cells. Therefore, therapeutic resources are essential to combat HIV infection and opportunistic infections.

In this study, the values of CD4^+^ T cell counts and viral load showed no association with the presence of PDDI. However, other authors observed an association of the event with a count less than 200 cells/µl^9^ ^,^ ^20^, and stated that DDI may affect patients' health.

A significant part of PDDI can be attributed to the fact that 100% of the ART in this study are metabolized by the cytochrome P450 enzyme system, acting as substrates (isozymes 1A2, 2A6, 2B6, 2C19, 2D6, and 3A4), inhibitors (isozymes 1A2, 2C8/9, 2C19,2D6, 2E1 and 3A4,) and inductors (isozymes 1A2, 2B6, 2C8/9, and 3A4)^4^. Theoretically, the co-administration of inhibitors or inducers may cause clinically significant changes in the pharmacokinetics and pharmacodynamics of other medicines (substrates). However, the rate and extent of metabolism are dependent on the expression of these isozymes, which can be influenced by genetic polymorphisms. It is estimated that genetics can explain 20-95% of the variability in therapeutic and toxic responses, including DDI^21^. In evaluating a specific DDI, it is important to note the relative inhibitory potential of the drug to the particular enzyme^22^.

Co-administration of ART and other medications can result in important changes in the serum levels, many of which are related to preventable adverse events. There is evidence that the HARRT, alone or combined with other medications, including action in the CNS, alters the CYP450 metabolism, which was observed in the present study, particularly in the PDDIs of moderate and higher severity^6^ ^-^ ^7^.

Regardless of the treatment time, 68.3% of PDDI were classified as moderate. Among these, 12.8% included ART and agents that act in the CNS (benzodiazepines, antidepressants, and neuroleptics) with a clinical impact of excessive sedation and confusion^6^ ^-^ ^7^. Whereas the users of these drugs are in the community, including developing industrial communities, this reaction can interfere with the quality of life and lead to negative outcomes. In these cases, a modification of therapy should be considered. For example, in the PDDI of diazepam and ritonavir, the use of a benzodiazepine such as lorazepam could prevent an increase in sedation effects^23^.

Although there has not been any PDDI reported between ART and other medicines and alcohol that resulted in the reduced serum levels of ART, the concomitant use of these with HARRT should not be neglected, particularly with the risk of occurrence of therapeutic failure^24^.

Despite the negative connotation of PDDI, it is necessary to consider that about two-fifths of the PDDI (41.7%) identified among the ART were beneficial, being used at the clinic as a therapeutic strategy. Evidence points to the protease inhibitor used in association with low-dose ritonavir (100 to 200 mg) that increased the suppression of viral replication. This dosage range indicates that ritonavir is a potent inhibitor of CYP3A4 isoenzyme; therefore, is used as an adjuvant with other protease inhibitors, except for nelfinavir. The protease inhibitor association with ritonavir provides higher serum levels, stable and long-lasting protease inhibitor, increasing its power of viral inhibition and reducing the occurrence of resistance mutations^25^. The concomitant use of tenofovir and atazanavir increases plasma levels by a minimum of 35% . The combination of tenofovir and darunavir raises the maximum plasma concentration by 16% and the minimum plasma concentration of darunavir and tenofovir by 24% and 37%, respectively. The association of ritonavir and tenofovir increases the exposure of tenofovir by 20%.

However, the medical prescription is the starting point for the use of the drug; the careful evaluation of the treatment regimen can help identify and predict the DDI and other potential problems related to medicines. The PDDI identified in this study, particularly those involving the CYP450 system, can be prevented through the appropriate drug dosage and patient clinical surveillance^8^. In this way, prescribers must analyze the aspects of patients to assess the risk-benefit of continued or combined medicines. In addition, the follow-up of the patients through domiciliary visits and conducting clinical and biochemical tests before and after the introduction of other medicines, will certainly contribute to a reduction of PDDI.

For nursing, despite the existence of institutionalized routines, it should interfere as assistance can prevent the occurrence of drug interactions, as well as to ensure safe practice when in hospital environments. The planning of the administration times and the intervals between the drugs are practical measures against the occurrence of PDDI^26^. The data from this study can be immediately applied in clinical practice, allowing nurses through knowledge of the pharmacokinetic and pharmacodynamics principles of PDDI in patients on antiretroviral treatment and its predisposing factors, leading them to identify possible adverse events related to its use.

The study has limitations. The evaluation of DDI was performed from a convenience sample of patients in ambulatory care, an aspect that limits the applicability from the findings to the general population, especially in patients assisted at other levels of the health care. An individual clinical evaluation of the risks and benefits of the DDI wasn't performed. Some combination therapies labeled as DDI may have been mandatory, due to the low tolerance of the patient to certain medications or unavailability of therapeutic alternatives with lower interactive potential.

Though the study was not designed to investigate the clinical impact of DDI, the findings, although limited are relevant to patients with HIV, especially for pointing out the most vulnerable groups to DDI. Additionally, the therapeutic regimens used in patients with HIV are very similar worldwide, especially after the advent of HAART. Generally, the prescription of these drugs is the responsibility of the Infectious Disease specialist, thus reducing the variability of the clinical practice.

Although there are contributions, particularly in Brazil for being the country with the second highest number of people infected with HIV and the pioneering nature of this study, it is important to point out the limitations of the research.

Potential drug interactions may have been underestimated given the lack of information about comorbidities, opportunistic infections, medications used in over-the-counter self-medication, practice mode, and the use of medicinal plants. We have not been able to evaluate the actual outcomes arising from drug interactions, which can be quite difficult, given that establishing cause and effect is complex particularly by the presence of polypharmacy, potentially interactive characteristics of many antiretroviral, and the eventuality of co-medication dose adjustment to overcome a certain PDDI.

## Conclusions

Risk factors were found for the occurrence of potential drug-drug interactions in the use of five or more medications and when the period of antiretroviral therapy exceeded six years. In the context of the pharmacoepidemiology of antiretroviral drugs, the moderate and higher severity potential drug interactions identified in this study, are events that not only affect the therapeutic response leading to toxicity in the central nervous and cardiovascular systems, but also can interfere in tests used for detection of HIV resistance to antiretroviral drugs.
